# Clinical features and outcome of influenza pneumonia in critically-ill immunocompromised patients

**DOI:** 10.1097/MD.0000000000032245

**Published:** 2022-12-09

**Authors:** Matthieu Raymond, Maëlle Martin, Pauline Lamouche-Wilquin, Gauthier Blonz, Paul Decamps, Maïté Agbakou, Luc Desmedt, Jean Reignier, Jean-Baptiste Lascarrou, Emmanuel Canet

**Affiliations:** a Service de Médecine Intensive Réanimation, CHU de Nantes, Nantes Université, Jean Monnet, France.

**Keywords:** Acute respiratory distress syndrome, immunocompromised, Influenza, intensive care unit, mortality

## Abstract

Immunocompromised subjects are at risk of severe viral infections which may require intensive care unit (ICU) admission. Data on the outcome of influenza pneumonia in critically-ill immunocompromised subjects are limited. We conducted a single-center observational study. All subjects admitted to the ICU for influenza pneumonia between 2016 and 2020 were included. The main objective was to compare the clinical features and outcome of critically-ill subjects with flu according to their immune status. 137 subjects (age 60 years-old, 58.4% male) were included, of whom 58 (42.34%) were intubated during the ICU stay. Forty-three (31.4%) subjects were immunocompromised. Immunocompromised subjects had a higher Charlson comorbidity index. In contrast, severity scores and hypoxemia at ICU admission, and ventilatory support during ICU stay were similar between the 2 groups. There was no difference in the rate of co-infections and ventilator-associated pneumonia between the 2 groups. Among intubated subjects, 10 (23.26%) immunocompromised subjects developed severe acute respiratory distress syndrome compared to 13 (13.83%) non-immunocompromised (*P* = .218). ICU mortality was 13.97%, with mortality being 3-times higher in immunocompromised subjects (25.58% vs 8.6%, *P* = .015). On multivariable analysis, immunocompromised status, higher age and lower arterial oxygen partial pressure/fraction of inspired oxygen were associated with an increased ICU mortality. Immunocompromised subjects with severe influenza pneumonia were more likely to develop severe acute respiratory distress syndrome and had a 3-fold increase in ICU mortality compared to non-immunocompromised subjects. Such difference was not explained by an increased rate of co-infections or nosocomial pneumonia, suggesting that influenza virus was by itself responsible of a more severe form of pulmonary disease in immunocompromised subjects.

**Key points**

**Current knowledge**

Immunocompromised patients are identified as a growing and frailty population at risk of severe influenza pneumonia which requires admission to the intensive care unit.

**What this paper contributes to our knowledge**

Immunocompromised subjects accounted for almost 1 to 3^rd^ of critically-ill subjects with influenza pneumonia. Although immunocompromised and non-immunocompromised subjects had similar respiratory parameters at ICU admission, comparable rates of intubation, co-infection and hospital acquired infection, ICU mortality of immunocompromised subjects with influenza infection was 3 times that of non-immunocompromised subjects.

## 1. Introduction

Seasonal influenza is an acute respiratory infection caused by influenza viruses. Influenza infection is a major cause of hospitalization, morbidity, and mortality worldwide, and a healthcare priority for the World Health Organization. All over the world, it is estimated that 3 to 5 millions of severe influenza cases are diagnosed, and that between 250,000 and 5,00,000 deaths from influenza infection occur annually^[1–3]^. In France, during the winter months of years 2018 to 2019, 10,723 hospitalizations and 1886 admissions to intensive care units (ICUs) were attributed to seasonal influenza^[4]^.

Some groups of subjects have been identified at high risk of developing severe illness and death, and therefore, are prioritized in vaccination policies^[1,5]^. These groups include the very young and elderly people, the pregnant women, the health care workers, and subjects with serious underlying medical conditions. Immunocompromised subjects are identified as a growing and frailty population at risk of influenza-related complications who require intensive care treatment. Several challenging issues are unique to this subject population including potential long-term exposure to steroids, sustained viral shedding, and high-risk of developing acute respiratory distress syndrome and opportunistic infections^[6,7]^. Nonetheless, data on the clinical features and outcome of seasonal influenza in immunocompromised subjects admitted to the ICU setting is limited (106).

Accordingly, we conducted an epidemiological study to compare the ICU management and outcomes of seasonal influenza between immunocompromised and non-immunocompromised subjects admitted to the ICU of French University-affiliated hospital. We hypothesized that immunocompromised subjects would experience a higher rate of ventilatory support, coinfections, ICU-acquired and fungal infections than non-immunocompromised subjects. Our findings may help intensivists for the management of influenza in immunocompromised subjects by providing relevant and updated information.

## 2. Methods

This retrospective study was approved by the ethics committee of the French Intensive Care Society (CE SRLF 21-107) on December 14, 2021 with a waiver for informed consent. The study is reported in compliance with the STROBE recommendations.^[8]^

### 2.1. Study design, setting, and population

We identified consecutive adults (≥18 years of age) admitted to the ICU of the Nantes University Hospital between November 1, 2016, and December 31, 2020, and registered in the regional electronic database of influenza infection monitoring (Cellule Régionale Pays de Loire). We cross-checked with subjects registered in the electronic hospital database with any of the codes for seasonal influenza in the international classification of diseases 10^th^ revision (ICD-10) coding system (J09, J10, J11). For subjects who had multiple admissions during the study period, only the 1^st^ admission was considered. Each medical file was reviewed by MR to confirm the diagnosis of seasonal influenza. Subjects were included if they had signs and symptoms of lower respiratory tract infection (fever, dyspnea, hypoxemia requiring oxygen, and pulmonary infiltrates on chest X-ray or computed tomography of the chest) at ICU admission and a positive polymerase in chain reaction for influenza virus on a naso-pharyngeal swab or a pulmonary sample (endotracheal aspiration or broncho-alveolar lavage). No other tests (rapid test, viral culture) were used for the diagnosis of influenza infection. Subjects without a positive polymerase in chain reaction for influenza virus were excluded.

### 2.2. Data collection

Data were extracted from the electronic medical records of the ICU (CERNER Millenium^®^, North Kansas city, MI). We obtained data for baseline subject characteristics, including demographics, comorbidities, chronic medications, onset of symptoms, and prior influenza vaccination. Subjects were defined as immunocompromised if they met 1 of the following criteria: hematopoietic stem cell transplantation, solid organ transplantation, human immunodeficiency virus infection, hematological malignancy or solid tumor newly diagnosed, progressing or in remission for < 5 years, steroids treatment for more than 3 months with a daily dose of prednisone of at least 7.5mg, and other immunosuppressive drugs. Physiological variables, laboratory data and radiographic findings (chest X-ray and computed tomography when available) on ICU admission were also reported. Disease severity was assessed using the simplified acute physiology score on day 1 after ICU admission. Acute Respiratory Distress Syndrome was defined according to the Berlin definition for subjects undergoing mechanical ventilation (invasive or noninvasive)^[9]^. Therapeutic regimens were reported including antiviral therapy (molecule, dose, and length of treatment). The life-sustaining therapies used during the ICU stay (high-flow oxygen, noninvasive ventilation, mechanical ventilation (MV), extracorporeal membrane oxygenation, vasopressors, and/or renal replacement therapy) were extracted from the electronic medical records. Co-infections (diagnosed within the 1^st^ 48 hours after hospitalization) and ICU-acquired infections (after 48 hours of hospitalization) were recorded. The diagnosis of bacterial infection was confirmed if subjects met both following criteria: microbiological identification of a pathogen and administration of antibiotic treatment. Ventilator-associated pneumonia was confirmed before antibiotics either by quantitative distal bronchoalveolar lavage cultures growing ≥ 10^4^ colony forming unit/mL or blind protected specimen brush distal growing ≥ 10^3^ colony forming unit/mL. The diagnosis of invasive pulmonary aspergillosis (IPA) was made if subjects met the criteria of putative or proven IPA according to the AspICU criteria^[10]^. Vital status was recorded at ICU discharge, hospital discharge, and 90 days after hospital discharge.

### 2.3. Objectives

The primary objective of the study was to compare the day-90 mortality of critically-ill subjects with seasonal influenza between immunocompromised and non-immunocompromised subjects.

The secondary objectives were to compare the ICU management, respiratory support, coinfections and ICU-acquired infections between immunocompromised and non-immunocompromised subjects.

### 2.4. Statistical analysis

Characteristics of subjects were described as frequencies and percentages for categorical variables and as means and standard deviations or medians and interquartile ranges for continuous variables. Continuous variables were compared using Student’s *t* test or Wilcoxon’s rank-sum test. Categorical variables are compared using Chi-square or Fisher’s exact test. Kaplan-Meier overall survival curves until Day 90 were computed, and were compared using log-rank tests. Baseline risk factors of death at Day 90 were assessed within the whole cohort using univariate and multivariate cox regression analyses. Baseline variables (i.e., obtained during the 1^st^ 24 hours in the ICU) included in the multivariate model were defined a priori, and no variable selection was performed. Candidates variables included in the multivariate model were: age, immunocompromised status, and arterial oxygen partial pressure (PaO_2_)/fraction of inspired oxygen (FiO_2_) ratio. A *P* value < 0.05 was considered statistically significant. Statistical tests were conducted using the R statistics program, version 3.5.0 (R Foundation for Statistical Computing, Vienna, Austria; www.R-project.org/) with R v3.5.1.

## 3. Results

### 3.1. Study population

During the study period, 137 subjects were admitted to the ICU for seasonal influenza, of whom 43 (31.4%) were immunocompromised and 94 (68.6%) were not immunocompromised. Table 1 reports their main features. No patients had a co-infection with SARS-CoV-2. The 3 most common causes of immunosuppression were chronic use of steroids (46.5%), solid tumors (39.5%), and hematological malignancies (37.2%) (Table 1 and Table S1, Supplemental Digital Content, http://links.lww.com/MD/I82).

**Table 1:** Baseline characteristics of the study participants.

	All patients (n = 137)	Immunocompromised patients (n = 43)	Non-immunocompromised patients (n = 94)	*P* value
**Demographic characteristics**				
Age, yr, mean +/- SD	60 (+/- 16.5)	64.77 (+/- 11.4)	58 (+/- 18.1)	.071
Male gender, n (%)	58.4%	62.8%	56.4%	.576
BMI, mean +/- SD	27.1 (+/- 7)	27.9 (+/- 8.8)	26.8 (+/- 6.1)	.43
Type of immunodepression, n (%)				/
* Immunosuppressive drugs or steroids*	20 (14.6%)	20 (46.5%)	0 (0%)	/
* Solid tumor*	17 (12.4%)	17 (39.5%)	0 (0%)	/
* Hematological malignancy*	16 (11.7%)	16 (37.2%)	0 (0%)	/
* Solid organ transplantation*	6 (4.4%)	6 (13.95%)	0 (0%)	/
* Primary immune deficiency*	1 (0.7%)	1 (2.33%)	0 (0%)	/
Other comorbidities, n (%)				
* Cardiovascular disease* *	76 (55.5%)	29 (67.4%)	47 (50%)	.085
* Chronic respiratory disease*	49 (35.8%)	14 (32.6%)	35 (37.2%)	.735
* Diabetes*	35 (25.6%)	7 (16.3%)	28 (29.8%)	.141
* Obesity*	35 (27.8%)	11 (29.7%)	24 (27%)	.923
* Chronic Kidney Disease*	20 (14.6%)	9 (20.9%)	11 (11.7%)	.193
Charlson comorbidity index, mean +/- SD	4 (+/- 2.6)	5.8 (+/- 2.4)	3.1 (+/- 2.3)	< .001
Performance status 0 - 2, n (%)	122 (89.1)	40 (90.9)	82 (88.2)	.774
Influenza vaccination	28 (23.9%)	8 (23.5%)	20 (24.1%)	1
**Characteristics at ICU admission**				
Time from first symptoms, d, mean +/- SD	5.3 (+/-4.5)	6 (+/- 5)	5 (+/- 4.3)	.309
Type of virus				
* Influenza A*	113 (82.5%)	33 (76.7%)	80 (85.1%)	.295
* Influenza B*	23 (16.8%)	10 (23.3%)	13 (13.8%)	
* Influenza A + B*	1 (0.7%)	0 (0%)	1 (1.1%)	
SAPS2, mean +/- SD	38.4 (+/-17.4)	42.1 (+/-16.4)	36.8 (+/-17.7)	.064
PaO_2_/FiO_2_ ratio, mean +/- SD	190.6 (+/-99.7)	186.4 (+/-110.8)	192.6 (+/-92.3)	.445
Antiviral treatment, n (%)	115 (83.9%)	37 (86.1%)	78 (83%)	.389
Antibiotic treatment, n (%)	129 (94.2%)	39 (90.7%)	90 (95.7%)	.258
**Respiratory support at ICU admission**				
Standard oxygen	77 (56.2%)	30 (69.77%)	47 (50%)	.048
Noninvasive ventilation	13 (9.49%)	3 (6.98%)	10 (10.65%)	.754
High Flow Oxygen	17 (12.41%)	2 (4.65%)	15 (15.96%)	.092
Invasive Mechanical Ventilation	30 (21.89%)	8 (18.6%)	22 (23.4%)	.658

BMI *=* Body mass index, FiO_2_ = fraction of inspired oxygen, PaO_2_ = arterial oxygen partial pressure, SAPS = simplified acute physiology score.

* *including hypertension*.

Immunocompromised subjects had more comorbidities than non-immunocompromised subjects. Influenza vaccination was reported in 23.9% of the subjects, without difference between immunocompromised and non-immunocompromised subjects. At ICU admission, subjects had a respiratory rate of 26 (±1.17)/min and a PaO_2_/FiO_2_ ratio of 190.6 (±16.9) without significant differences between the 2 groups.

### 3.2. Clinical features of influenza and ICU management

Subjects were admitted to the ICU 5.3 (±1.17) days after the onset of symptoms. Table 2 provides details about the treatments used and complications observed in the ICU. Oseltamivir was administered at ICU admission to 122 (89%) subjects, at a dose of 150 mg twice daily during 7.1 (±0.7) days. Only 32 (23.4%) subjects received oseltamivir within 48 hours after the onset of symptoms. There was no difference in antiviral treatment between immunocompromised and non-immunocompromised subjects. Nearly 1 quarter of the subjects received corticosteroids during the ICU for the management of septic shock, bronchospasm, or needed hydrocortisone replacement therapy for long-term exposure to corticosteroids.

**Table 2:** Process of care and outcomes.

	All patients (n = 137)	Immunocompromised patients (n = 43)	Non-immunocompromised patients (n = 94)	*P* value
**ICU Management**				
Invasive ventilation during ICU stay, n (%)	58 (42.3%)	18 (41.9%)	40 (42.6%)	1
ARDS	51 (37.2%)	17 (39.5%)	34 (36.2%)	.851
* Severe ARDS, n (%*)	23 (16.8%)	10 (23.3%)	13 (13.9%)	.218
Neuromuscular blockers, n (%)	32 (23.4%)	12 (27.9%)	20 (21.3%)	.526
Prone positioning, n (%)	20 (14.6%)	10 (23.3%)	10 (10.6%)	.068
ECMO, n (%)	4 (2.9%)	1 (2.3%)	3 (3.2%)	1
Vasopressors, n (%)	63 (46%)	21 (48.8%)	42 (44.7%)	.788
* Duration, d, mean +/- SD*	4.3 +/- 5.3	4.2 +/- 5.2	4.4 +/- 5.3	.563
Acute Kidney Injury, n (%)	97 (70.8%)	32 (74.4%)	65 (69.2%)	.686
* KDIGO 3 AKI, n (%*)	30 (30.9%)	9 (28.1%)	21 (32.3%)	.91
* Renal remplacement therapy, n (%*)	11 (8%)	3 (7%)	8 (8.5%)	1
**Outcomes**				
Lenght of stay in ICU, d, mean +/- SD	9,2 (+/- 11)	8.8 (+/- 9.4)	9.3 (+/- 11.6)	.826
Ventilator-free days at D28, d, mean +/- SD	20.1 +/- 11.3	17.8 +/- 12.9	21.3 +/- 10.4	.247
ICU mortality (n = 136)	19 (14%)	11 (25,6%)	8 (8,6%)	.015
Hospital mortality (n = 129)	23 (17,8%)	14 (35%)	9 (10,2%)	.001
Mortality at d 90 (n = 103)	24 (23,3%)	15 (42,9%)	9 (13,2%)	.001

ARDS = acute respiratory distress syndrome, ECMO = extracorporeal membrane oxygenation, ICU = intensive care unit, KDIGO = kidney disease improval global outcomes, SD = standard derivation.

Fifty (36.5%) subjects had a documented bacterial co-infection, with *Stretptococcus pneumoniae* and *Staphylococcus aureus* being the most common isolated pathogens (Table S2, Supplemental Digital Content, http://links.lww.com/MD/I83 and Table S3, Supplemental Digital Content, http://links.lww.com/MD/I84). *Pseudomonas aeruginosa* was isolated in 3 subjects; all of them were immunocompromised. The rate of co-infection was 41.5% in non-immunocompromised subjects and 25.6% in immunocompromised subjects (*P* = .09).

During the ICU stay, 58 (42.3%) subjects were intubated 0.6 (±1.4) day after ICU admission, and invasive mechanical ventilation (IMV) was implemented during 14.7 (±15.1) days without difference between immunocompromised and non-immunocompromised subjects. Among the 58 subjects treated with IMV, 51 (37.2%) met the criteria of acute respiratory distress syndrome (ARDS), of whom 23 (16.8%) developed severe ARDS. Immunocompromised subjects had a higher occurrence of severe ARDS compared to non-immunocompromised subjects (23.3% vs 13.8%, *P* = .218). Overall, 13 (22.4%) subjects had a diagnosis of ventilator associated pneumonia, with similar figures between immunocompromised and non-immunocompromised subjects (22.2% vs 22.5%, *P* = 1).

Two (1.5%) fungal infections and 1 (0.7%) parasitic infection were diagnosed. One invasive pulmonary aspergillosis occurred in a non-immunocompromised subject treated with extracorporeal membrane oxygenation. One *Pneumocystis* pneumonia was reported in a liver transplant recipient, and 1 leishmaniasis associated with hemophagocytic syndrome was diagnosed in a subject with chronic myelomonocytic leukemia.

### 3.3. Outcomes and factors associated with mortality

**Figure 1.**
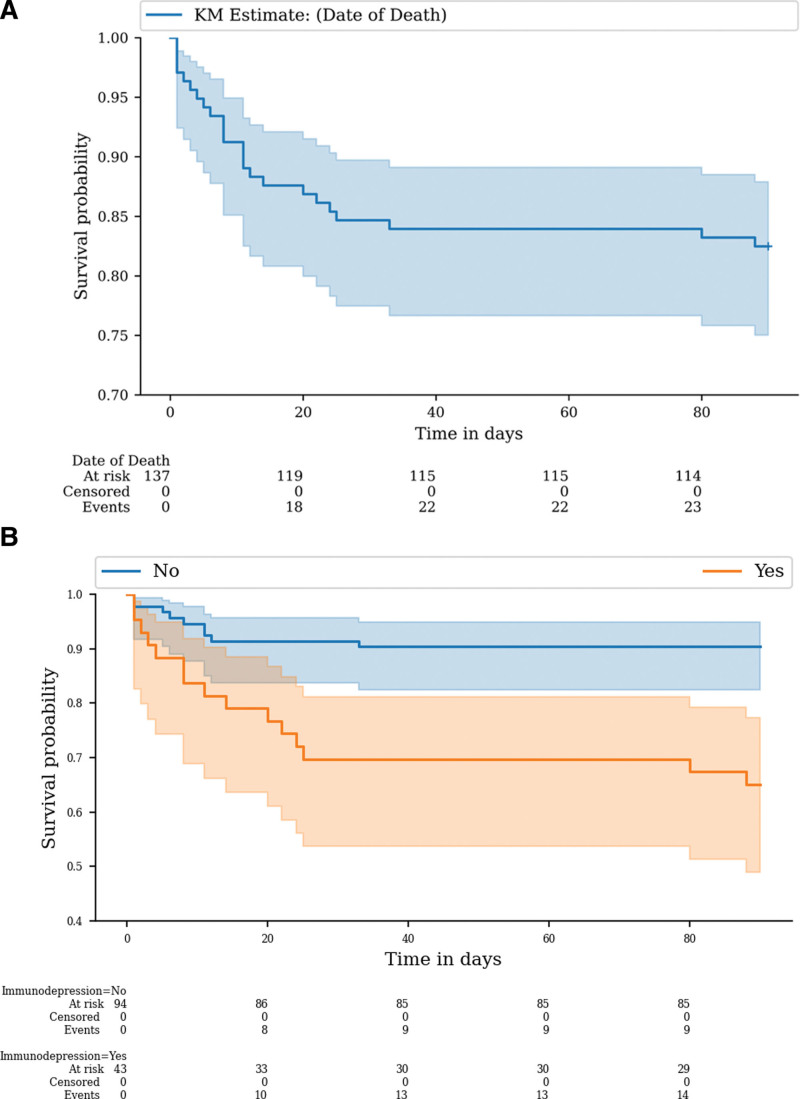
Day-90 mortality in the overall population (A) and in the groups with and without immunosuppression (B).

During the study period, 19 (14%) subjects died in the ICU and 23 (17.8%) died during the same hospital stay. Details on the mortality rate per year are provided in the supplementary appendix (Table S4, Supplemental Digital Content, http://links.lww.com/MD/I85). Immunocompromised subjects had a 3-fold increase in ICU and hospital mortalities compared to non-immunocompromised subjects (Table 2 and Fig. 1). By univariate analysis, age, immunocompromised status, comorbidities, poor performance status, and high simplified acute physiology score were associated with an increased risk of mortality. By multivariable analysis, immunocompromised status, higher age and lower PaO_2_/FiO_2_ ratio at admission were associated with ICU mortality (Table 3).

**Table 3:** Univariate and multivariable analysis of factors associated with ICU mortality.

	Univariate analysis		Multivariable analysis	
	Hazard ratio	*P* value	Hazard ratio	*P* value
Age†	1.04 (1.00–1.09)	.03	1.05 (1.01–1.08)	.008
Immunocompromised status	2.97 (1.19–7.41)	.019	3.69 (1.68–8.09)	.001
Performance status††	2.19 (1.19–4.02)	.011		
Charlson comorbidity index††	1.30 (1.07–1.59)	.009		
PaO_2_/FiO_2_ ratio††	0.99 (0.98–1.00)	.06	0.99 (0.98–0.99)^+^	.028
SAPS2††	1.03 (1.01–1.06)	.002		
Documented bacterial co-infection	1.13 (0.45–2.80)	.79		
Acute Kidney Injury	3.75 (0.48–28.79)	.2		

FiO_2_ = fraction of inspired oxygen, HR = hazard ratio, PaO_2_ = arterial oxygen partial pressure, SAPS = simplified acute physiology score.

† Risk for each 1-year increase,

†† Risk for each 1-point increase.

## 4. Discussion

### 4.1. Key findings

We used a regional electronic registry of influenza infection and the ICD-10 coding system to identify ICU subjects with influenza infection and to compare the clinical features and outcomes of immunocompromised and non-immunocompromised subjects. We found that immunocompromised subjects accounted for almost 1 to 3^rd^ of critically-ill subjects with influenza infection, were older, and had more comorbidities compared to non-immunocompromised subjects. Furthermore, although immunocompromised and non-immunocompromised subjects had similar respiratory parameters at ICU admission and comparable rates of intubation, the occurrence of severe ARDS in immunocompromised subjects was nearly twice that of non-immunocompromised subjects. There were no differences in rates of co-infections, opportunistic infections, and ventilator-associated pneumonia between immunocompromised and non-immunocompromised subjects. Finally, the ICU mortality of immunocompromised subjects with influenza infection was 3 times that of non-immunocompromised subjects.

### 4.2. Comparison to previous studies

In high-income countries, the population of immunocompromised subjects is steadily growing^[11]^ as a result of increased life-expectancy, improved diagnostic methods, and major therapeutic advances^[12–16]^. Immunosuppression increases the risk and severity of infections^[17,18]^ which may require ICU management. Acute respiratory failure is the 1^st^ cause of ICU admission in immunocompromised subjects ^[14,19,20]^ and viruses accounted for 15% to 20% of severe community-acquired pneumonia in such subjects ^[21]^. In a study conducted in Spain between 2009 and 2015, 12.5% of ICU subjects with influenza A (H1N1) pneumonia were immunocompromised^[22]^. In our experience, 31.4% of ICU subjects with influenza infection were immunocompromised. Although this difference might be explained by discrepancies in vaccination coverage or ICU admission policies, we hypothesize that because our study was conducted more recently, it is a witness of the growing number of subjects living with an underlying cause of immunosuppression. We found that immunocompromised subjects were older and had more comorbidities that non-immunocompromised subjects, in line with previous studies^[22,23]^.

Co-infections are frequently reported in critically-ill subjects with influenza although their incidence and impact on clinical outcomes remain controversial^[24–26]^. A recent longitudinal study reported an increased incidence of bacterial co-infection over a 7-year period (from 11.4% to 23.4%), and identified immunosuppression as being associated with an increased risk of co-infection (OR 1.4 [1.1–1.9])^[27]^. In addition, co-infection was an independent predictor of ICU mortality^[27]^. Our study also reported a high rate of co-infection (36.5%). However, we did not find an increased risk of co-infection in immunocompromised subjects. Our data should be interpreted cautiously due to the limited number of subjects and because 90% of them had empirical antibiotics started at ICU admission, even though similar findings have been reported by other authors^[22]^. Furthermore, the diagnosis of co-infection was not associated with a higher mortality in our study. Martin-Loeches et al analyzed the association between each pathogen and mortality and found that co-infections with *Aspergillus*, *Pseudomonas aeruginosa*, and methicillin-sensitive *Staphylococcus aureus* were associated with significant mortality^[27]^. In our study, the main bacteria identified in co-infections were *Streptococcus pneumoniae*, *Staphylococcus aureus*, and *Streptococcus pyogenes*. Therefore, such differences in the local epidemiology may lead to different outcomes.

Influenza has been identified as a risk factor of invasive pulmonary aspergillosis, with an incidence of 19% in the intensive care unit, and up to 32% among immunocompromised subjects ^[28]^. Moreover, Schauwvlieghe et al reported a high mortality in subjects with influenza-associated invasive pulmonary aspergillosis (51%)^[28]^. This was not our experience with only 1 non-immunocompromised subject diagnosed with invasive pulmonary aspergillosis. The exact incidence of invasive pulmonary aspergillosis in critically-ill subjects remains a matter of debate. Indeed, a recent multicenter study conducted by Coste et al among 524 ICU subjects with influenza pneumonia, only 10 (1.9%) subjects had a diagnosis of putative or proven invasive pulmonary aspergillosis^[29]^. Likewise, a recent pilot trial failed to demonstrate the benefit posaconazole for the prevention of invasive pulmonary aspergillosis in critically-ill subjects with influenza infection^[30]^. These conflicting results underline the limits of the current definitions for invasive pulmonary aspergillosis in this setting and the need for additional research.

Ventilator-associated pneumonia (VAP) is a common complication of invasive mechanical ventilation in the ICU. Although its impact on mortality is unclear, it is associated with extended duration of mechanical ventilation and ICU length of stay^[31]^. Garnacho-Montero et al reported a VAP incidence of 7.5% in ICU subjects with influenza without difference between immunocompromised and non-immunocompromised subjects ^[22]^. In another large multicenter observational study of critically-ill immunosuppressed subjects, 13.1% of subjects with influenza infection had ICU-acquired pneumonia^[32]^. In our study, 22.2% of immunocompromised subjects had a diagnosis of VAP compared 22.5% of non-immunocompromised subjects (*P* = 1).

Immunocompromised subjects are at higher risk of more severe seasonal influenza. A study conducted in the United States demonstrated that immunocompromised subjects with influenza had a higher a risk of being hospitalized, being admitted to the ICU and treated with invasive mechanical ventilation, had more pulmonary infiltrates on chest X-ray, and had extended viral shedding compared to non-immunocompromised subjects ^[6]^. In our study, immunocompromised subjects with influenza infection had almost a 2-fold increased risk of developing severe ARDS and an ICU mortality 3-times that of non-immunocompromised subjects. These findings are in line with previous studies^[1,22,27,33]^.

### 4.3. Study implications

The findings from our study imply that immunocompromised subjects are a noteworthy population, accounting nowadays for more than 30% of ICU subjects with severe influenza pneumonia. Such subjects are at higher risk of developing severe ARDS and dying in the ICU compared to non-immunocompromised subjects, supporting a broad ICU admission policy. Moreover, our findings imply that, although common, the rates of co-infections, fungal infections and VAP did not differ between immunocompromised and non-immunocompromised subjects. Therefore, a strategy targeting infectious complications associated with influenza pneumonia, such as antibiotic prophylaxis or antifungal prophylaxis is unlikely to improve survival in immunocompromised subjects. We hypothesize that the severity of influenza pneumonia in immunocompromised subjects is mainly related to the damage of the lungs induced by the influenza virus itself. Finally, improving the prognosis of influenza pneumonia in immunocompromised subjects should rely on both prioritizing vaccination and developing more effective antiviral drugs.

### 4.4. Strengths and limitations

This study has a number of strengths. First, we used both the ICD-10 coding system and a regional electronic registry to identify subjects with seasonal influenza. This minimized potential bias related to the retrospective selection of the study subjects. Second, we conducted a detailed review of each medical file to compare the clinical features, complications and outcomes of immunocompromised versus non-immunocompromised subjects. Thus, we provide new and updated data for guiding the management of the vulnerable population of immunocompromised subjects. Third, we obtained detailed information on the infectious complications associated with influenza to analyze their impact on subjects’ outcome according to their immunocompromised status, an area rarely explored in previous ICU studies.

Our study also has several limitations. First, the retrospective design implies information bias with a possibility of missing data. Second, the study was conducted in a single institution, where the case mix may have significantly influenced our findings. Nonetheless, we conducted this study in the ICU of a large university-affiliated center, and our results should therefore apply to similar settings in high-income countries. Third, although ICD-10 discharge coding combined with regional electronic registry has strong reliability for diagnosing seasonal influenza, we cannot exclude that we missed some subjects and studied a particular cohort of subjects with more easily diagnosed and, perhaps, more severe and prolonged seasonal influenza pneumonia. Fourth, we did not routinely collect biomarkers such as procalcitonin or C-reactive protein. Therefore, we cannot comment on their value in this setting. Finally, the ICU management of the subjects and the treatments delivered were not standardized and left at the discretion of the attending physician. This led to potential heterogeneity in practices and prevented us from evaluating how treatments may have affected subject outcomes. However, we collected data over a short period of time (2016-2020) during which ICU practices and influenza treatment remained unchanged.

## 5. Conclusion

In conclusion, critically-ill immunocompromised subjects with seasonal influenza had a higher risk of severe ARDS and death than non-immunocompromised subjects. However, immunocompromised subjects had a similar rate of co-infections, fungal infections and ventilator associated pneumonia compared to non-immunocompromised subjects, suggesting that the more severe lung damage was explained by the influenza virus itself rather than associated complications. In addition to prioritizing influenza vaccination for immunocompromised subjects, further studies are needed to assess if more effective antiviral drugs could translate into better subjects’ outcomes.

## Acknowledgements

Emmanuel Canet takes responsibility for (is the guarantor of) the content of the manuscript, including the data and analysis.

## Author contributions

**Conceptualization:** Matthieu Raymond, Jean Reignier, Emmanuel Canet.

**Data curation:** Matthieu Raymond, Maëlle Martin, Pauline Lamouche-Wilquin, Gauthier Blonz, Paul Decamps, Maïté Agbakou, Luc Desmedt, Jean Reignier, Jean-Baptiste Lascarrou.

**Formal analysis:** Matthieu Raymond, Jean Reignier, Emmanuel Canet.

**Investigation:** Maëlle Martin, Pauline Lamouche-Wilquin, Gauthier Blonz, Paul Decamps.

**Methodology:** Matthieu Raymond, Jean-Baptiste Lascarrou, Emmanuel Canet.

**Supervision:** Emmanuel Canet.

**Writing – original draft:** Matthieu Raymond.

**Writing – review & editing:** Jean Reignier, Emmanuel Canet.

## Supplementary Material


