# Supplementary material for: Clinical features and outcome of influenza pneumonia in critically-ill immunocompromised patients
Source: Medicine (Baltimore). 2022 Dec 9;101(49):e32245. doi: 10.1097/MD.0000000000032245 (PMC9750560; doi:10.1097/MD.0000000000032245)
Supplement: Supplementary file 1 [file medi-101-e32245-s001.pdf]

Supplemental Digital Content

e-Table 1: Causes of immunosuppression

	**n (%)**
**Hematological malignancy**	16 (37.21%)
Allogeneic stem cell transplantation	3
Lymphoma	6
Myeloma	4
Acute leukemia	1
Myelodysplasia	1
Chronic myelomonocytic leukemia	1
**Solid tumor**	17 (39.53%)
Lung	3
Breast	3
Liver	2
ENT	2
Others[1]	7
**Solid organ transplantation**	6 (13.95%)
Kidney	5
Liver	1
**Immunosuppressive drugs or steroids**	20 (46.51%)
Treatment to prevent organ rejection	9
Autoimmune or inflammatory disease	7
Others[2]	4
**Primary immune deficiency**	1 (2.33%)

*ENT : Ear, Nose and Throat
[1] Melanoma : n = 2, bladder : n = 1, kidney : n = 1, neuroendocrine : n = 1, prostate : n = 1, thymoma : n = 1
[2] Asthma : n = 3, multiple sclerosis : n = 1
