# Supplementary material for: Clinical features and outcome of influenza pneumonia in critically-ill immunocompromised patients
Source: Medicine (Baltimore). 2022 Dec 9;101(49):e32245. doi: 10.1097/MD.0000000000032245 (PMC9750560; doi:10.1097/MD.0000000000032245)
Supplement: Supplementary file 2 [file medi-101-e32245-s002.pdf]

Supplemental Digital Content

e-Table 2: Microbiological investigations

	Overall (n=137)	Immunocompromised (n=43)	Non-immunocompromised (n=94)	p-value
**Number of respiratory samples collected, n (%)**	86 (62,77%)	27 (62,79%)	59 (62,77%)	1
Sputum culture, n (%)	32 (37,20%)	8 (29,63%)	24 (40,68%)	0.721
Bronchoalveolar lavage cultures or blind protected specimen brush distal, n (%)	51 (59,3%)	19 (70,37%)	32 (54,24%)	
Endotracheal aspiration, n (%)	1 (1,16%)	0 (0%)	1 (1,69%)	
Pleural puncture, n (%)	2 (2,33%)	0 (0%)	2 (3,39%)	
Samples collected before antibiotics, n (%)	17 (19,77%)	4 (14,81%)	13 (22,03%)	0.565
Number of samples with a positive culture, n (%)	27 (31,40%)	7 (25,93%)	20 (33,90%)	0.617
**Positive antigenuria (n=114), n (%)**	14 (12,28%)	2 (5,88%)	12 (15%)	0.224
Streptococcus pneumoniae	13 (11.4%)	2 (5.88%)	11 (13.75%)	0.223
Legionnella pneumophila	1 (0.88%)	0 (0%)	1 (1.25%)	0.241
**Positive blood culture (n=129), n (%)**	13 (10,08%)	4 (10,26%)	9 (10%)	1
