# Supplementary material for: Clinical features and outcome of influenza pneumonia in critically-ill immunocompromised patients
Source: Medicine (Baltimore). 2022 Dec 9;101(49):e32245. doi: 10.1097/MD.0000000000032245 (PMC9750560; doi:10.1097/MD.0000000000032245)
Supplement: Supplementary file 3 [file medi-101-e32245-s003.pdf]

Supplemental Digital Content

e-Table 3: Co-infections and ventilator associated pneumonia

	Overall (n=137)	Immunocompromised (n=43)	Non-immunocompromised (n=94)	p-value
**Bacterial co-infections, n (%)**	50 (36,5%)	11 (25,6%)	39 (41,5%)	0.087
Streptococcus pneumoniae, n (%)	20 (14.6%)	4 (9.3%)	15 (17%)	
Staphylococcus aureus, n (%)	9 (6.6%)	3 (7%)	6 (6.4%)	
Streptococcus pyogenes, n (%)	5 (3.6%)	1 (2.3%)	4 (4.3%)	
Haemophilus influenzae, n (%)	3 (2.2%)	0 (0%)	3 (3.2%)	
Pseudomonas aeruginosa, n (%)	3 (2.2%)	3 (7%)	0 (0%)	
Stenotrophomonas maltophila, n (%)	2 (1.5%)	0 (0%)	2 (2.1%)	
Legionnella pneumophila, n (%)	1 (0.7%)	0 (0%)	1 (1.1%)	
Other gram-positive cocci, n (%)*	5 (3.7%)	1 (2.3%)	4 (4.3%)	
Other gram-negative bacile⁺, n (%)	5 (3.7%)	1 (2,3%)	4 (4.3%)	
**Viral co-infections, n (%)**	5 (3.7%)	2 (4.7%)	3 (3.2%)	0.649
**Fungal infections, n (%)**	2 (1.5%)	1 (2.3%)	1 (1.1%)	0.531
**Ventilator associated pneumonia (n = 58), n (%)**	13 (22,4%)	4 (22,2%)	9 (22,5%)	1
Pseudomonas aeruginosa, n (%)	4 (6.9%)	2 (11.1%)	2 (5%)	
Enterococcus species, n (%)	3 (5.2%)	1 (5.6%)	2 (5%)	
Staphylococcus aureus, n (%)	2 (3.5%)	0 (0%)	2 (5%)	
E. Coli, n (%)	2 (3.5%)	1 (5.6%)	1 (2.5%)	
Other gram-negative bacile°, n (%)	7 (12.1%)	0 (0%)	7 (17.5%)	
Other gram-positive cocci#, n (%)	1 (1.7%)	1 (5.6%)	0 (0%)	

* : *Streptococcus mitis : n = 2, Streptococcus aggalactiae : n = 1, Enterococcus faecalis : n = 1, gram-positive cocci with missing identification : n = 1*

⁺ : *Escherichia coli : n = 2, Moraxella catarrhalis : n = 2, Serratia marcescens : n = 1*

# : *Staphylococcus haemolyticus : n = 1*

° : Enterobacter cloaque : n = 2, Burkholderia spp : n = 1, Citrobacter Koseri : n = 1, Hafnia alvei : n = 1, Klebsiella oxytoca : n = 1, Proteus mirabilis : n = 1
