# Supplementary material for: Clinical features and outcome of influenza pneumonia in critically-ill immunocompromised patients
Source: Medicine (Baltimore). 2022 Dec 9;101(49):e32245. doi: 10.1097/MD.0000000000032245 (PMC9750560; doi:10.1097/MD.0000000000032245)
Supplement: Supplementary file 4 [file medi-101-e32245-s004.pdf]

Supplemental Digital Content

e-Table 4: Mortality rate per year

Year of ICU admission	All patients n (%)	Immunocompromised patients n (%)	ICU mortality n (%)
2016	6 (4.4%)	3 (50%)	1 (16.7%)
2017	40 (29.2%)	12 (30%)	4 (10%)
2018	48 (35%)	16 (33.3%)	7 (14.6%)
2019	29 (21.2%)	7 (24.1%)	6 (20.7 %)
2020	14 (10.2%)	5 (35.7%)	1 (7.1%)
